# Live birth after fresh versus frozen single blastocyst transfer (Frefro-blastocyst): study protocol for a randomized controlled trial

**DOI:** 10.1186/s13063-017-1993-5

**Published:** 2017-06-05

**Authors:** Daimin Wei, Yun Sun, Jiayin Liu, Xiaoyan Liang, Yimin Zhu, Yuhua Shi, Zi-Jiang Chen

**Affiliations:** 1 Center for Reproductive Medicine, Shandong Provincial Hospital Affiliated to Shandong University, Key Laboratory of Reproductive Endocrinology, Shandong University, Ministry of Education, and National Research Center for Assisted Reproductive Technology and Reproductive Genetics, Jinan, China; 2 Center for Reproductive Medicine, Ren Ji Hospital, School of Medicine, Shanghai Jiao Tong University, Shanghai Key Laboratory for Assisted Reproduction and Reproductive Genetics, Shanghai, China; 3 Department of Obstetrics and Gynecology, First Affiliated Hospital of Nanjing Medical University, Nanjing, China; 4 Reproductive Medicine Center, the Sixth Affiliated Hospital of Sun Yat-sen University, Guangzhou, China; 5 Department of Reproductive Endocrinology, Women’s Hospital, School of Medicine, Zhejiang University, Hangzhou, China; 6 157 Jingliu Road, Jinan, 250000 China

**Keywords:** Single embryo transfer, Blastocyst, Singleton live birth, Randomized controlled trial

## Abstract

**Background:**

Multiple pregnancies are one of the major safety concerns of in vitro fertilization (IVF) due to the increased risk of maternal and neonatal complications. Single embryo transfer is the most effective way to reduce the risk of multiple pregnancies. Selection of the embryo and optimization of the implantation environment are crucial to retain the success rate when the number of transferred embryos is diminished. Fresh embryo transfer with supra-physiological levels of hormones has been suggested to have an adverse effect on implantation. Elective frozen embryo transfer has been suggested to result in a higher rate of live birth than fresh embryo transfer. However, there is still a lack of evidence from randomized clinical trials comparing the efficacy and safety between frozen and fresh single blastocyst transfers.

**Methods/design:**

We are conducting a randomized controlled trial in women aged 20–35 undergoing their first cycle of IVF with or without intracytoplasmic sperm injection. After ovarian stimulation with a gonadotropin-releasing hormone antagonist protocol, women who obtain four or more embryos on day 3 of the embryo culture are randomized into two parallel groups: a single fresh blastocyst transfer group and a single frozen blastocyst transfer group (all blastocysts vitrified and a deferred frozen blastocyst transfer). The primary outcome is singleton live birth.

**Discussion:**

The results of this study will provide evidence for the efficacy and safety of the strategy of elective frozen single blastocyst transfer in women with a good prognosis.

**Trial registration:**

Chinese Clinical Trial Registry, ChiCTR-IOR-14005405. Registered on 30 Oct 2014.

**Electronic supplementary material:**

The online version of this article (doi:10.1186/s13063-017-1993-5) contains supplementary material, which is available to authorized users.

## Background

Since the birth of the first baby conceived by in vitro fertilization (IVF) in 1978, IVF has been increasingly used worldwide [1]. Historically, to compensate for the low rate of implantation of individual embryos and achieve acceptable pregnancy rates, multiple embryos were transferred at a time. As the implantation rate continues to improve with the advancements in embryo culture, concerns have increasingly been raised on the risk of multiple pregnancies. The number of embryos transferred has been reconsidered [2, 3]. By reducing the number of embryos transferred to two, the incidence of high-order pregnancies has been dramatically decreased without compromising the pregnancy rate [4]; however, the risk of twin pregnancies remains unchanged [5]. Elective single embryo transfer (eSET) is the most effective approach to reduce the risk of twin pregnancies [3, 6]. eSET is recommended to patients with a good prognosis: age <35 years, more than one top-quality embryo available for transfer, first or second treatment cycle [5]. However, the adoption of the eSET policy has been slow because of concerns about the decreased pregnancy rate after single embryo transfer compared with double embryo transfer [2, 3, 7]. Selection of the embryo and optimization of the uterine environment are crucial to retain the success rate.

In order to improve the selection of the embryo, extended culture to the blastocyst stage has been increasingly utilized. Blastocysts are suggested to have greater implantation potential than cleavage-stage embryos [8]. Single blastocyst transfer has resulted in significantly higher rates of pregnancy and delivery than single cleavage-stage embryo transfer [9]. To optimize the uterine environment, frozen embryo transfer may be a better choice than fresh embryo transfer. Controlled ovarian hyperstimulation and the resulting supra-physiological hormones have been suggested to have a detrimental effect on oogenesis, implantation of embryos [10], endometrial development [11] and frequency of uterine contraction [12], and perhaps perinatal outcomes [13]. Frozen embryo transfer allowing the recovery of supra-physiological hormones and the shedding of the exposed endometrium may provide a better physiological environment for embryo implantation. On the other hand, developments in the technology of cryopreservation, especially the introduction of vitrification, has greatly increased the survival rate of the embryo after thawing and enabled a non-inferior pregnancy rate after frozen embryo transfer compared with fresh embryo transfer [14]. Recently, a freeze-all and delayed frozen embryo transfer policy has been advocated and has achieved wide popularity [15–17]. In patients with polycystic ovary syndrome (PCOS), by transferring cleavage-stage embryos, we had observed that frozen embryo transfer resulted in a higher rate of live birth, a lower rate of ovarian hyperstimulation syndrome (OHSS), and a higher birth weight of the singleton, but a higher risk of preeclampsia compared with fresh embryo transfer [18]. However, there is still a lack of evidence as to whether a frozen blastocyst transfer could also provide any advantage over a fresh blastocyst transfer. A randomized trial is warranted to test the efficacy and safety of the strategy of single frozen blastocyst transfer. This study is a multicenter randomized controlled trial comparing the efficacy and safety of single frozen blastocyst transfer with single fresh blastocyst transfer in women with a good prognosis.

## Methods/design

### Study population

#### Inclusion criteria

The inclusion criteria are as follows:

1. Women aged ≧20 and <35 years
2. Women with regular menses (defined as a spontaneous cycle length of 21–35 days)
3. Women who are undergoing their first cycle of IVF or intracytoplasmic sperm injection (ICSI)
4. Women with ≧4 day-3 embryos suitable for transfer.

#### Exclusion criteria

The exclusion criteria are as follows:

1. Women who have been diagnosed with congenital or acquired uterine abnormalities such as uterine malformation (uterus unicornis, septate uterus, or duplex uterus), adenomyosis, submucous myoma, or intrauterine adhesion
2. Women who are to undergo preimplantation genetic diagnosis/preimplantation genetic screening
3. Women who are to undergo frozen embryo transfer before randomization such as those who are scheduled to be treated for hydrosalpinx, who have elevated progesterone during controlled ovarian hyperstimulation, or who have high risk of severe OHSS
4. Women with medical conditions that contraindicate assisted reproductive technology and/or pregnancy, such as uncontrolled hypertension or known symptomatic heart disease; poorly controlled type 1 or type 2 diabetes mellitus; undiagnosed liver disease or dysfunction (based on serum liver enzyme test results); renal disease or abnormal serum renal function; severe anemia; history of deep venous thrombosis, pulmonary embolus, or cerebrovascular accident; history of (or suspected) cervical carcinoma, endometrial carcinoma, or breast carcinoma.

### Study intervention

#### Screening

**Fig. 1:**
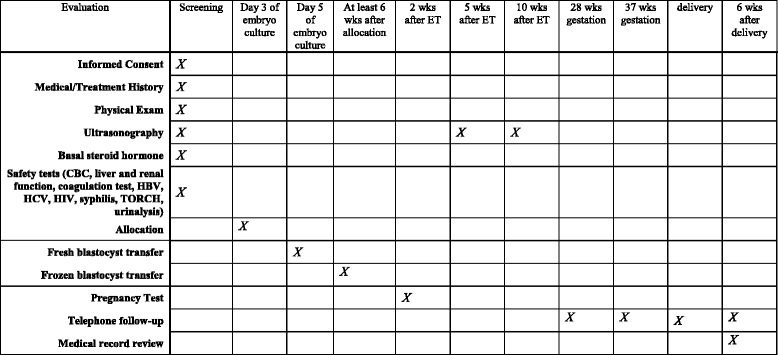
SPIRIT figure: the schedule of enrollment, interventions, and assessments

**Fig. 2:**
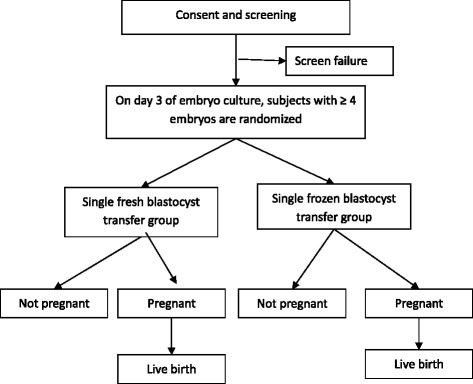
Flow chart of subjects

At the screening visit, written informed consent will be obtained from the couples. Previous medical history and current medication status are reviewed with the standardized case report forms. A physical examination and transvaginal ultrasound scan are performed. Laboratory measurements including basal sex hormone tests, safety assays such as liver function, renal function, hepatitis virus, HIV, syphilis, coagulation, blood routine, and urine routine are performed in the local labs of the study sites. All of the subjects and their partners are karyotyped to exclude couples with abnormal karyotype. A diagnostic hysteroscopy is performed in patients who are suspected to have an abnormal uterine cavity. A schedule of enrollment, interventions, and assessment is provided in the Standard Protocol Items: Recommendations for Interventional Trials (SPIRIT) figure (Fig. 1). The flow chart of this study is given in Fig. 2.

#### Controlled ovarian hyperstimulation

The gonadotropin-releasing hormone (GnRH) antagonist protocol is used for ovarian stimulation in all subjects. Recombinant follicle-stimulating hormone (rFSH, Puregon; MSD Organon, Oss, The Netherlands) is started on days 1–3 of the menstrual cycle. The dose of rFSH is adjusted according to ovarian response monitored by hormone tests and transvaginal ultrasound scan. Human menopausal gonadotropin can be added at the discretion of local investigators. GnRH antagonist ganirelix (MSD Organon, Oss, The Netherlands) at a daily dose of 250 μg is administered when there is at least one follicle ≥12 mm in mean diameter until the trigger day (including the trigger day). Human chorionic gonadotropin (hCG) at a dose of 4000–10,000 IU will be administered when at least two follicles are ≥18 mm in mean diameter.

#### Oocyte retrieval, in vitro fertilization, and embryo culture

Transvaginal ultrasound-guided follicle aspiration is performed 34–36 h after hCG injection by experienced physicians. Oocytes are inseminated by IVF or ICSI according to the quality of sperm. If the total number of motile sperm is below 5 million, ICSI is performed. Mixed IVF and ICSI as half ICSI (50% oocytes are inseminated by IVF and 50% oocytes by ICSI) or rescue ICSI (if the second polar body is absent in more than 60% of oocytes after IVF, these oocytes will be re-inseminated by ICSI) are also allowed.

On day 3 of the embryo culture, the quality of embryos is assessed by morphological criteria mainly based on the number and regularity of blastomeres as well as the percentage of fragmentation. Subjects with four or more embryos that are suitable for transfer are randomly assigned to the fresh or frozen blastocyst transfer group. Blastocyst culture is performed for all embryos of the randomized subjects.

#### Randomization

Block randomization is used to assign subjects to treatment groups with a 1:1 ratio. The randomization is stratified by study site. The sequence of randomization has been generated by biostatisticians in data coordinator center with SAS software version 9.2 (SAS Institute, Cary, NC, USA). The original sequence is safely kept by the staff in the data coordinator center, and it has been input into the online central randomization system by these staff members, who are not involved in enrolling subjects. The online sequence is not accessible to any investigators or study coordinators. If a subject fulfills the enrollment criteria, the authorized investigator or study coordinator will login the password-protected account to get the assignment for her. After randomization, both subjects and investigators are informed about the assignments.

#### Single fresh blastocyst transfer

On day 5 of embryo culture, women who are assigned to the fresh blastocyst transfer undergo a single blastocyst transfer. Luteal phase support with vaginal progesterone gel (Crinone, Merck Serono, Darmstadt, Germany) 90 mg/day and oral dydrogesterone 10 mg twice daily is started from the day of oocyte retrieval and continued until 12–15 days after embryo transfer when serum hCG is measured to determine pregnancy. If pregnancy is achieved, luteal phase support will be continued until 10 weeks’ gestation.

#### Single frozen blastocyst transfer

On day 5 of embryo culture, women who are assigned to the frozen blastocyst transfer group have their blastocysts vitrified. Luteal phase support that started from the day of oocyte retrieval with the same regimen as the fresh blastocyst transfer group will be stopped after randomization. At least 4 weeks later, endometrium preparation is performed with a natural cycle regimen or hormone replacement cycle regimen, at the discretion of local investigators.

For the natural cycle regimen, ovulation is determined by ultrasound monitoring. Local investigators decide whether to use hCG for ovulation triggering according to their clinical routine. Luteal phase support is started from the ovulation day with oral dydrogesterone 10 mg three times daily. A single frozen-thawed blastocyst will be transferred 5 days after ovulation. If the patient is pregnant, luteal phase support will continue until 10 weeks’ gestation.

For the hormone replacement cycle regimen, the endometrium is prepared with oral estradiol valerate at a dose of 4–8 mg daily started on day 1 to day 3 of the menstrual cycle. Vaginal progesterone gel (Crinone, Merck Serono) 90 mg/day and oral dydrogesterone 10 mg twice daily are added when the endometrial thickness reaches 7 mm or more. A single frozen-thawed blastocyst will be transferred 5 days after progesterone initiation. Estradiol valerate at the dose for endometrium preparation will be continued until the day of the hCG test. If pregnancy is achieved, estradiol valerate is stopped gradually at 8 to 9 weeks of gestation; vaginal progesterone gel and oral dydrogesterone is continued until 10 weeks of gestation.

#### Outcome and outcome assessments

The primary outcome is singleton live birth. The secondary outcomes include conception, clinical pregnancy, ongoing pregnancy, pregnancy loss, live birth, moderate and severe OHSS, ectopic pregnancy, pregnancy and perinatal complication, birth weight, neonatal complication, and other adverse events.

Regarding conception, serum β-hCG is measured to determine pregnancy 12 days after embryo transfer. Conception is defined with the result of serum β-hCG ≥10 mIU/mL.

Regarding clinical pregnancy, if a conception is achieved, a transvaginal ultrasound scan is performed 20 days later. Clinical pregnancy is defined as detection of a gestational sac in the uterine cavity. An ectopic pregnancy is defined as one in which the embryo implants at any site other than the endometrial lining of the uterine cavity.

For an ongoing pregnancy, a transvaginal ultrasound scan is repeated at 11–12 weeks’ gestation. Ongoing pregnancy is defined as detection of a viable fetus with fetal heartbeat.

During pregnancy, women are contacted by telephone call every 3 months to inquire about adverse events. Pregnancy loss is defined as pregnancies that eventuate in a spontaneous abortion or therapeutic abortion that occurred during the pregnancy.

At delivery, obstetrical and perinatal complications as well as neonatal information are obtained with reference to obstetric medical records and neonatal medical records. A singleton live birth is defined as the delivery of one newborn ≥28 weeks’ gestation with heartbeat and breath. A live birth is defined as the delivery of any number of newborns ≥28 weeks’ gestation with heartbeat and breath.

At 6 weeks after delivery, postpartum complications and neonatal complications are followed up by telephone call.

Moderate OHSS is diagnosed when ultrasonographic ascites is present in addition to abdominal distension and discomfort with or without nausea, vomiting, and/or diarrhea. Severe OHSS is diagnosed when there is clinical evidence of ascites and/or hydrothorax or breathing difficulties with or without hemoconcentration, coagulation abnormalities, and diminished renal function.

Adverse events are any untoward or unfavorablemedical occurrences associated with the subject’s participation in the research, whether or not considered related to the study intervention. Serious adverse events are events that are temporally associated with the subject’s participation in research that meet any of the following criteria: death, life-threatening, severely or permanently disabling, requiring in-patient hospitalization or prolongation of existing hospitalization, pregnancy loss after 20 weeks gestation, neonatal death up to 6 weeks after delivery, congenital anomaly or birth defect, or any events deemed as serious by the local principal investigator.

### Data analysis

#### Sample size calculation

Based on the retrospective data from our IVF clinic, the live birth rate following single fresh blastocyst transfer in patients younger than 35 years is about 50%. It was assumed that an absolute difference of 10% in live birth rate will be of clinical significance. We aim to test a difference of 10% of live birth rate between treatment groups (i.e., 50% in the fresh group and 60% in the frozen group) at a significance level of 0.01 with a statistical power of 90%. The minimal sample size calculated is 735 for each group. In consideration of a dropout rate of 10%, we will enroll 817 subjects in each group.

#### Data collection

Data are collected with a standard case report form developed in the web-based data entry system with real-time logical and range checking. Data are de-identified before being input into the database. Regular study site monitor and database checking are performed to ensure the accuracy of data collected.

#### Data analysis plan

The primary analysis will use an intent-to-treat approach to examine differences in the singleton live birth rate in the two treatment arms using the Pearson chi-square test. Safety parameters and secondary efficacy parameters, such as pregnancy rate, OHSS rate, and other rates, will be analyzed using the Pearson chi-square test. A secondary per-protocol analysis will be performed according to the actual treatment that subjects received. Continuous data were expressed as mean ± standard deviation with a Wilcoxon rank sum test for testing between-group differences. Categorical data were represented as frequency and percentage; differences in these measures between treatment groups were assessed by chi-square analysis, with Fisher’s exact test for expected frequencies less than 5.

The flow chart of this study is given in Fig. 2, and the SPIRIT checklist is included as Additional file 1.

## Discussion

This is a study comparing the efficacy and safety of single frozen blastocyst transfer with single fresh blastocyst transfer in women with a good prognosis. We plan to enroll 1618 subjects from more than 20 academic IVF centers in China. The enrollment began in August 2016. At the time of manuscript preparation, more than 300 subjects have been enrolled. The result of this large multicenter randomized trial will provide level I evidence for the strategy of single frozen blastocyst transfer in patients with a good prognosis.

Elective embryo cryopreservation followed by frozen embryo transfer, i.e., the freeze-all strategy, has attracted increasing attention and has been considered as a potential innovation of IVF treatment [15]. However, there are still great gaps in the literature to illuminate the risk/benefit ratio of this strategy, which includes multiple steps of treatment [19]. Four randomized trials compared frozen versus fresh embryo transfer [18, 20–22]. However, the stage and the number of embryos transferred varied among studies. Embryos were frozen by the slow-freezing method at 2PN stage in the earlier studies [20–22]. In a recent study in women with PCOS, embryos were vitrified at the cleavage stage, and two embryos were transferred [18]. There are several striking differences in uterine condition and embryo characteristics between cleavage-stage embryo transfer and blastocyst transfer. It was observed that uterine contractility had been significantly decreased at the time of fresh blastocyst transfer compared with that at the time of fresh cleavage-stage embryo transfer, and high-frequency uterine contraction was suggested to negatively impact the outcome of IVF via the dislocation of the embryo or disruption to the interaction between embryo and endometrium [23, 24]. Additionally, there were concerns regarding epigenetic modifications during blastocyst culture. A meta-analysis pooling six observational studies demonstrated that singleton pregnancies following blastocyst transfer were associated with higher risks of preterm birth and congenital anomalies compared with those following cleavage-stage embryo transfer [25]. Whether these differences necessarily translate to less superiority of frozen blastocyst transfer over fresh blastocyst transfer is unknown.

In women with a good prognosis, i.e., those who are defined as young and undergoing their first IVF cycle and obtaining four or more day-3 transferrable embryos in this study, eSET is recommended to reduce the risk of multiple pregnancies. However, the adoption of this policy has been slow due to concerns about the diminished pregnancy rate. Extending embryo culture to the blastocyst stage to improve embryo selection has been demonstrated as an effective measurement. Whether replacing the well-selected embryo into a more physiological uterine environment could further increase the chance of live birth of eSET is of great clinical significance. This study is expected to provide a reliable answer.

## Trial status

The enrollment of this study is ongoing at the time of manuscript submission.

## Additional file

Additional file 1: SPIRIT checklist. (PDF 80 kb)

## Abbreviations

eSET: Elective single embryo transfer
GnRH: Gonadotropin-releasing hormone
hCG: Human chorionic gonadotropin
ICSI: Intracytoplasmic sperm injection
IVF: *In vitro* fertilization
OHSS: Ovarian hyperstimulation syndrome
PCOS: Polycystic ovary syndrome
rFSH: Recombinant follicle-stimulating hormone
